# An analysis of health facility services readiness for non-communicable diseases in 8 LMICs in the universal health coverage era

**DOI:** 10.34172/hpp.43175

**Published:** 2024-12-30

**Authors:** Shailender Singh, Meenakshi Kaul, Chandrashekhar J Rawandale

**Affiliations:** Symbiosis Law School, Noida, Symbiosis International Deemed University, Pune, India

**Keywords:** Cardiovascular diseases, Diabetes, Respiratory diseases, Service readiness

## Abstract

**Background:**

The readiness of health facility services for non-communicable diseases (NCDs) is a critical aspect of global health infrastructure. NCDs, such as cardiovascular diseases, chronic respiratory diseases, and diabetes, pose significant challenges to public health systems worldwide. This study assesses health facility services readiness for NCDs in 8 low- and middle-income countries (LMICs).

**Methods:**

The data is collected using stratified random sampling method with a sample size of 7606 health facilities, the study assesses the health facility services readiness index for general and disease–specific health services by using the survey data from service provision assessment (SPA) between 2015 to 2021 for eight countries under the study. This service readiness index represents the percentage of items considered essential for providing general and specific health services issued by the World Health Organisation.

**Results:**

The mean values of the service readiness index at 95% confidence interval are 56.2%, 37.7%, 35.4%, and 36.5% for the general health services, diabetes, cardiovascular, and respiratory diseases, respectively. These results show substantial variations range from 1% to 20 % in the service readiness index by health facility types in the countries of this study. Overall, public facilities have achieved a higher service readiness index score and, thus, have demonstrated a greater level of preparation in providing general and disease–specific health services for chronic non–communicable diseases.

**Conclusion:**

A substantial number of health facilities in these countries are not adequately prepared to care for chronic NCDs. More investment in critical health infrastructure is urgently needed to strengthen the capacity of health systems in the countries of this study. The investment should focus on achieving universal health coverage (UHC) goals vis-à-vis reducing the burden of premature mortality from chronic diseases.

## Introduction

 Universal health coverage (UHC) is the current architecture driving the post-2015 global health targets. UHC is considered as the central pillar of Sustainable Development Goals (SDGs) and an essential element for ensuring equity in access to health care at a global scale.^1^ Investment into well-equipped health care systems is a desirable and necessary condition for attaining UHC goals. Regardless of the existing scarce resources common to low- and middle-income countries (LMICs), these countries must adequately and continuously provide a wider range of health services that are affordable, accessible, and effective for improvement in health outcomes. The different aspects of health-system design for successful transitions to the UHC are described elsewhere,^2^ and excess mortality attributed to the utilization of low-quality health services in the UHC era in LMICs is estimated.^3^ While research from different fields has provided perspectives to the literature regarding the UHC debate, there is a dearth of an investigation into the health system’s capacity to provide services for chronic non-communicable diseases (NCDs) in the UHC era.

 Providing high-quality care requires the readiness of the services of health facilities.^4^ Services readiness of health facilities depends on the level of investment in the country’s health system. However, many contemporary discussions on UHC tend to focus on the expansion of health insurance coverage (the demand for care) by ignoring the strength of health facilities to offer high-quality health services to the population (the supply side). Although some studies have examined the strength of health systems to provide essential services,^5^ access to primary care in Mongolia,^6^ essential medicine to treat NCDs in Uganda,^7^ health services for diabetes and cardiovascular diseases in Bangladesh,^8^ there has not been any multi-country study of services readiness for diabetes, cardiovascular diseases, and respiratory diseases in LMICs in the UHC era.

 Recently, the growing burden of NCDs has constituted a substantial portion of the global mortality and morbidity burden. NCDs, which include type 2 diabetes, cardiovascular diseases, cancer, and respiratory diseases, among others, affect people of different economic and age groups.^9^ Importantly, roughly 80% of the NCDs burden is attributed to LIMCs.^10^ From the global estimates of 53 million mortality figures, 35 million are attributed to NCDs, and a substantial part of these figures occurred in LMICs.^11^ Across the globe, NCDs have accounted for 44% of premature mortality. The death toll attributed to these conditions has roughly doubled the mortality rates from all other major causes of death combined.^9^ In addition, the burden of NCDs is only projected to increase. Available estimates suggest an increase in the NCDs burden in LMICs by almost 17% and up to 27% in some regions of the World, including Africa.^12^

 Therefore, the score of the service readiness index is increasingly used for disease-specific studies.^8,9^ In assessing the capacity of health facilities to provide disease-specific services; the service readiness index is often employed as a generalizable metric for many reasons. First, it indicates the strength of health facilities to offer basic care and its ability to provide timely responses to emergency health crises in the population.^13^ Secondly, it helps identify differences that may exist within a country’s health system in terms of resource allocation. Thirdly, it can serve as a benchmark for estimating and comparing the efficiency of the health system in processing health care resources inputs into output, usually referred to as health outcome.^5^

 However, the objective of this study is to assess health facility services readiness for diabetes, cardiovascular and respiratory diseases in Afghanistan, Bangladesh, Congo Democratic Republic, Haiti, Malawi, Nepal, Senegal, and Tanzania in the UHC era.

## Material and Methods

 The sample size of this paper comprised all the health facilities that were involved in the Service Provision Assessment (SPA) surveys conducted by the Demographic and Health Survey (DHS) program between 2015 and 2021.^14^ The data for the assessment of services readiness for chronic diseases is available for 8 LMICs: Afghanistan, Bangladesh, Congo Democratic Republic, Haiti, Malawi, Nepal, Senegal, and Tanzania. The datasets come from samples that are national representatives of the countries’ health systems. However, data from Afghanistan come from only one region of the country. Across these countries, the sample size involved public facilities, public not-for-profit/NGO facilities, and private for-profit facilities. However, in the Congo Democratic Republic and Malawi, some facilities are owned by faith groups. Furthermore, mixed ownership of some health facilities is reported in Haiti.

###  Data and sample size

 This study used the most recent wave of SPA survey data collected between 2015 to 2021 in eight LMICs. The SPA survey is typically conducted by the DHS program, with funds received from the United States Agency for International Development (USAID) as the principal donor. The SPA survey collects and documents nationally representative data that is made freely available for research purposes upon request. The facility-level survey collects data from health facilities; unless stated otherwise, it used to be a census of health facilities across the country. It collects nationally representative data from all types of formal sector health facilities at regional and national levels.

 Initially, the SPA surveys focused primarily on maternal and child health. However, from the last decade to date, their scope has been broadened to capture data on the general capacity of health facilities and the quality of health care. From 2007 onwards, the inventory questionnaire is used to collect information on a wider range of service coverages and the capacity of the facility to provide care. Table 1 presents the countries of this study and the details of the data.

**Table 1:** Demographic and health survey profile of the studied countries, 2013-2019

**Countries**	**Year of service provision assessment **	**Survey coverage**	**No. of facilities surveyed**
Afghanistan	2020-2021	Regional	160
Bangladesh	2019	National	1,600
Congo Democratic Republic	2019-20	National	1,414
Haiti	2019-20	National	1,033
Malawi	2015-16	National	1,060
Nepal	2017	National	992
Senegal	2021	National	454
Tanzania	2016-17	National	1,200

Source: Demographic and Health Surveys.

**Table 2:** General service readiness index and each domain score by facility type

**General service coverage**	**Public facilities (%)**	**Private not for profit (%) **	**Private for-profit (%)**	**Mixed (%)**	**Faith-based (%)**	**Others (%)**	**Total (%)**
Basic amenities							
Power supply	2,618 (60.0)	506 (11.6)	874 (20.1)	252 (5.77)	53 (1.21)	65 (1.49)	4,368 (57.4)
Functional Generator	1,465 (45.1)	404 (12.4)	786 (24.2)	550 (17.0)	13 (0.40)	33 (1.02)	3,251 (92.0)
Source of water	1,684 (59.0)	357 (12.5)	503 (17.6)	200 (7.00)	53 (1.86)	59 (2.07)	2,856 (37.4)
Communication	929 (47.3)	276 (14.1)	471 (24.0)	204 (10.4)	46 (2.34)	38 (1.94)	1.964 (25.8)
Computer	2,190 (57.8)	417 (11.0)	726 (19.2)	383 (10.1)	51 (1.35)	24 (0.63)	3,791 (49.8)
Functional ambulance	1,165 (57.0)	223 (11.3)	404 (19.8)	180 (8.81)	24 (1.17)	48 (2.35)	2,044 (26.9)
Mean domain score	54.4	12.2	20.8	9.85	8.33	1.57	48.2
Basic equipment							
Digital blood pressure	962 (46.4)	310 (14.9)	510 (24.6)	192 (9.25)	48 (2.31)	53 (2.55)	2,075 (27.6)
Manual blood pressure	4,156 (63.8)	600 (9.21)	1,067 (16.4)	626 (9.61)	29 (0.30)	39 (0.60)	6,517 (86.6)
Adult scale	4,296 (43.7)	648 (9.45)	1,126 (16.5)	641 (9.38)	56 (0.92)	63 (1.50)	6,831 (89.8)
Child scale	2,771 (56.6)	355 (8.40)	608 (14.4)	436 (10.3)	25 (0.60)	31 (0.73)	4,226 (55.5)
Infant scale	2,634 (67.2)	314 (8.02)	555 (14.2)	381 (9.72)	16 (0.41)	17 (0.43)	3,917 (51.5)
Stethoscope	4,642 (63.7)	669 (9.18)	1,193 (16.4)	663 (9.10)	53 (0.73)	63 (0.86)	7,284 (95.8)
Thermometer	4,287 (62.8)	648 (9.49)	1,129 (16.5)	645 (9.45)	56 (0.82)	63 (0.92)	6,828 (89.8)
Light source	2,383 (58.6)	391 (9.60)	791 (19.4)	440 (10.8)	36 (0.88)	28 (0.69)	4,069 (53.5)
Mean domain score	57.9	9.78	17.3	9.70	0.87	1.04	68.8
Standard precaution							
Sharps container	3,818 (66.3)	526 (9.14)	835 (14.5)	463 (8.05)	49 (0.85)	64 (1.11)	5,755 (75.7)
Waste receptacle	2,716 (65.3)	336 (8.07)	661 (15.9)	415 (9.97)	16 (0.38)	17 (0.41)	4,161 (87.1)
Running water	3,377 (60.8)	571 (10.3)	953 (17.2)	532 (9.6)	56 (1.00)	61 (1.10)	5,550 (73.0)
Hand washing soap	3,137 (60.9)	527 (10.2)	880 (17.1)	500 (9.70)	50 (0.97)	59 (1.14)	5,153 (67.7)
Latex gloves	3,889 (62.9)	588 (9.50)	1,032 (16.7)	563 (9.10)	52 (0.84)	63 (1.02)	6,187 (81.3)
Gowns	2,263(54.7)	446 (10.8)	792 (17.6)	545 (13.2)	38 (0.92)	54 (1.31)	4,137 (55.0)
Medical masks	1,509 (55.2)	346 (12.7)	525 (19.2)	269 (9.84)	32 (1.17)	53 (1.94)	2,734 (36.3)
Eye goggles	349 (47.7)	114 (15.6)	150 (20.5)	80 (10.9)	18 (2.26)	20 (2.74)	731 (9.72)
Guidelines for standard precautions	1,113 (59.6)	220 (11.8)	287 (15.4)	186 (15.3)	31 (1.66)	32 (1.71)	1,869 (24.6)
Mean domain score	59.3	10.9	17.1	10.6	1.12	1.39	56.7
Diagnostic capacity							
Haemoglobin test	1,903 (44.4)	349 (8.15)	583 (13.6)	187 (4.37)	50 (1.17)	60 (1.40)	4,283 (55.0)
Blood glucose test	1,758 (48.5)	442 (12.2)	886 (24.5)	503 (13.9)	19 (0.52)	14 (0.39)	3622 (68.5)
Urine chemistry test	2,996 (75.5)	587 (14.8)	1,028 (25.9)	539 (13.6)	23 (0.58)	11 (0.28)	3,969 (75.0)
Syphilis test	1,713 (52.0)	397 (12.0)	702 (21.3)	453 (13.7)	17 (0.52)	15 (0.45)	3,297 (62.3)
Tuberculosis test	421 (64.5)	31 (4.75)	85 (13.0)	112 (17.2)	3 (0.46)	1 (0.15)	653 (26.8)
Renal function	264 (58.4)	36 (7.96)	99 (21.9)	49 (10.8)	3 (0.67)	0 (0.00)	452 (18.5)
Mean domain score	57.2	10.0	20.0	12.3	0.65	0.45	51.0
General service readiness index	57.2	10.7	18.8	10.6	2.7	1.11	56.2

**Table 3:** Service readiness index for diabetes and each domain score by facility type

**Service coverage for diabetes**	**Public facilities (%)**	**Private not for profit (%)**	**Private for-profit (%)**	**Mixed (%)**	**Faith-based (%)**	**Others (%)**	**Total** **(%)**
Diagnosis and management	664 (53.3)	124 (10.0)	346 (29.2)	113 (9.06)	0 (0.0)	0 (0.0)	1,247 (18.0)
National guidelines for diabetes	553 (53.7)	112 (11.2)	175 (17.6)	133 (13.4)	8 (0.91)	11 (1.11)	992 (22.3)
Mean domain score	53.5	10.6	23.4	11.2	0.46	0.56	20.2
Adult scale	4,296 (43.7)	648 (9.45)	1,126 (16.5)	641 (9.38)	56 (0.92)	63 (1.50)	6,831 (89.8)
Stadiometer	2,909 (66.6)	376 (8.61)	579 (13.3)	436 (10.0)	25 (0.57)	40 (0.92)	4,365 (57.4)
Digital blood pressure	962 (46.4)	310 (14.9)	510 (24.6)	192 (9.25)	48 (2.31)	53 (2.55)	2,075 (27.6)
Manual blood pressure	4,156 (63.8)	600 (9.21)	1,067 (16.4)	626 (9.61)	29 (0.30)	39 (0.60)	6,517 (86.6)
Mean domain score	60.1	10.5	17.7	9.56	1.03	1.40	65.4
Blood glucose test	1,758 (48.5)	442 (12.2)	886 (24.5)	503 (13.9)	19 (0.52)	14 (0.39)	3622 (68.5)
Urine protein test	1,222 (65.4)	120 (6.42)	323 (17.3)	198 (10.6)	2 (0.10)	3 (0.16)	1,868 (28.0)
Urine glucose test	1,125 (63.7)	118 (6.68)	326 (18.5)	192 (10.9)	1 (0.06)	4 (0.23)	1,766 (26.5)
Mean domain score	59.2	8.51	20.1	11.8	0.23	0.26	41.0
Injectable insulin	495 (37.9)	171 (13.1)	355 (27.2)	275 (21.1)	2 (0.15)	5 (0.40)	1,305 (17.7)
Glibenclamide	454 (36.1)	177 (14.1)	382 (30.3)	207 (16.4)	18 (1.42)	21 (1.67)	1,259 (17.1)
Metformin	491 (37.6)	198 (15.1)	437 (33.4)	153 (11.7)	7 (0.54)	23 (1.76)	1,307 (17.7)
Injectable glucose	1,692 (52.4)	383 (11.9)	640 (19.8)	451 (14.0)	37 (1.14)	29 (0.90)	3,232 (43.9)
Mean domain score	41.0	13.6	27.7	15.8	1.08	1.18	24.1
Service readiness index for diabetes	53.5	43.0	22.3	48.4	0.7	0.85	37.7

**Table 4:** Service readiness index for cardiovascular diseases and each domain score by facility type

**Service coverage for cardiovascular diseases **	**Public facilities (%)**	**Private not for profit (%)**	**Private for-profit (%)**	**Mixed (%)**	**Faith-based (%)**	**Others (%)**	**Total (%)**
Diagnosis and management	938 (61.0)	155 (10.1)	339 (22.0)	106 (6.89)	0 (0.0)	0 (0.0)	1538 (23.7)
National guidelines for cardiovascular diseases	704 (60.0)	127 (10.8)	185 (1.66)	121 (10.3)	16 (1.36)	21 (1.79)	1174 (22.4)
Mean domain score	60.5	10.5	11.8	8.60	0.68	0.90	22.1
Adult scale	4296 (43.7)	648 (9.45)	1,126 (16.5)	641 (9.38)	56 (0.92)	63 (1.50)	6831 (89.8)
Stadiometer	2909 (66.6)	376 (8.61)	579 (13.3)	436 (10.0)	25 (0.57)	40 (0.92)	4365 (57.4)
Digital blood pressure	962 (46.4)	310 (14.9)	510 (24.6)	192 (9.25)	48 (2.31)	53 (2.55)	2075 (27.6)
Manual blood pressure	4156 (63.8)	600 (9.21)	1067 (16.4)	626 (9.61)	29 (0.30)	39 (0.60)	6517 (86.6)
Mean domain score	55.1	10.5	17.7	9.56	1.03	1.40	65.4
Amlodipine	596 (40.7)	215 (14.7)	447 (30.6)	192 (13.1)	3 (0.20)	10 (0.68)	1463 (19.9)
Atenolol (Beta-Blocker)	648 (37.3)	293 (16.8)	556 (32.0)	208 (12.0)	7 (0.40)	27 (1.55)	1739 (23.6)
Captopril	557 (43.9)	198 (15.6)	371 (29.2)	123 (9.69)	7 (0.55)	14 (1.10)	1270 (17.2)
Nifedipine	408 (34.2)	214 (17.9)	366 (30.6)	179 (15.0)	5 (0.41)	21 (1.76)	1193 (18.5)
Thiazide	405 (40.5)	171 (17.1)	258 (25.8)	119 (11.9)	23 (2.3)	24 (2.4)	1000 (13.6)
Mean domain score	39.3	19.9	29.6	12.3	0.77	1.50	18.6
Service readiness index for cardiovascular diseases	51.6	13.6	19.7	10.2	0.83	1.27	35.4

**Table 5:** Service readiness index for respiratory diseases and each domain score by facility type

**Service coverage for** **respiratory diseases**	**Public facilities (%)**	**Private not for profit (%)**	**Private for-profit (%)**	**Mixed (%)**	**Faith-based (%)**	**Others (%)**	**Total (%)**
Diagnosis and management	1690 (73.0)	149 (6.43)	363 (15.7)	117 (5.05)	0 (0.0)	0 (0.0)	2319 (35.7)
National guidelines for respiratory diseases	785 (62.0)	128 (10.1)	186 (14.7)	134 (10.6)	11 (0.89)	23 (1.62)	1267 (24.5)
Mean domain score	67.5	8.27	15.2	7.83	0.45	0.81	30.1
Adult weighing scale	4296 (43.7)	648 (9.45)	1126 (16.5)	641 (9.38)	56 (0.92)	63 (1.50)	6831 (89.8)
Stadiometer	2909 (66.6)	376 (8.61)	579 (13.3)	436 (10.0)	25 (0.57)	40 (0.92)	4365 (57.4)
Digital blood pressure	962 (46.4)	310 (14.9)	510 (24.6)	192 (9.25)	48 (2.31)	53 (2.55)	2075 (27.6)
Manual blood pressure	4156 (63.8)	600 (9.21)	1067 (16.4)	626 (9.61)	29 (0.30)	39 (0.60)	6517 (86.6)
Mean domain score	55.1	10.5	17.7	9.56	1.03	1.40	65.4
Salbutamol Inhaler	1196 (54.7)	241 (11.0)	464 (21.2)	229 (10.5)	28 (1.28)	28 (1.28)	2186 (29.7)
Simvastatin	71 (3.25)	54 (16.8)	154 (47.8)	37 (11.5)	1 (0.31)	5 (1.55)	322 (4.37)
Beclomethasone	161 (27.3)	113 (19.2)	213 (36.2)	96 (16.3)	3 (0.51)	3 (0.51)	589 (7.80)
Mean domain score	28.4	15.7	35.1	12.8	0.7	1.11	14.0
Service readiness index for respiratory diseases	50.3	11.5	22.7	10.1	0.72	1.11	36.5

 Notably, the data was collected using a stratified random sampling method. Generally, all missing values are not included in the analysis, and thus, the sample size included 7606 health facilities, drawn from the formal health sector of these countries. Specifically, the number of facilities with service coverage for diabetes and respiratory diseases included 5155 and 5093 health facilities. However, facilities with service coverage of cardiovascular diseases have no missing values in the dataset.

###  Data analysis

 Predominantly, this study analysed the facility level service readiness from five broad perspectives – a public, private not-for-profit, private for-profit, mixed, and faith-based facilities. In addition, clinics and dispensaries are given under other classifications. This ensures full representation of all types of health facilities representing the health systems of the countries of this study. The present study employed the World Health Organization’s (WHO’s) manual of Service Readiness and Assessment as a standard framework for the analysis of the data.^15^ Explicitly, the WHO’s standard manual has four domains for assessing general service coverage – supply of basic amenities, supply of basic equipment, compliance to standard precautions for infection prevention, and diagnostic capacity. Moreover, service-specific coverage for diabetes, cardiovascular, and respiratory diseases is also assessed using the same standard manual issued by the WHO.^15^ Furthermore, the service coverage index for providing both diagnosis and management of diabetes, cardiovascular and respiratory diseases is assessed by using facility type as the unit of stratification. Table S1 (see Supplementary file 1) shows the details of each service coverage domain. Thus, the score for each domain is computed as the mean availability of the indicator used to gauge the service as a proportion within that domain. Ultimately, the mean of all the domain scores is computed and expressed for general service coverage, diabetes, cardiovascular diseases, and respiratory disease-specific service coverage. All the analysis of the data is performed using the STATA version 15.1 statistical software package.

## Results

 This paper established panel data for 8 LMICs that reported data on service assessment, as shown in Table 2. The mean domain scores of general service coverage at 95% confidence interval are 48.2, 68.8, 56.7, and 51.0 for the supply of basic amenities, basic equipment, standard precaution, and diagnostic capacity. Altogether, private for-profit facilities had lower mean domain scores compared to public facilities across the five domains. Basic amenities have the lowest mean domain scores. Among the items listed under standard precaution, eye goggles have the lowest supply of 731 (9.72%), followed by the availability of medical masks at 2,734 (36.3%). Thus, the undersupply of these items may lead to a quicker spread of nosocomial infections among health care workers. Though a significant number of health facilities have sharps containers for efficient waste disposal at 5,755 (75.7%), however, only 1,869 (24.6%) of the health facilities had guidelines for standard precautions. The renal function test had the lowest availability mean score, followed by the tuberculosis test under the diagnostic capacity of health facilities. Overall, considering the four domains, the general service readiness index is 56.2. Apparently, the service readiness index is relatively low for private for-profit health facilities at 20.0 compared to public health facilities at 57.2.

Table 3 shows the service readiness index specific to diabetes. Of the 5155 health facilities that are involved in providing diabetic care, only 1247 (18.0%) diagnose and manage diabetes. The level of diagnosis and management of diabetes is quite lower in private for-profit health facilities at 346 (29.2%) compared to public health facilities at 664 (53.3%). The private not-for-profit health facilities or health facilities managed by non-governmental organizations and those with mixed ownership had a low level of coverage in terms of diagnosis and management of diabetes at 124 (10.0%) and 113 (9.06%). Similarly, only 992 (22.3%) had national guidelines for diabetes management. The same applies to the level of compliance with standard national guidelines for diabetes management between private health facilities at 175 (17.6%) and public health facilities at 553 (53.7%). Health facilities under mixed ownership had a higher level of compliance to national guidelines for diabetes management at 133 (0.91%) relative to private not-for-profit health facilities at 112 (11.2%).

 Availability of basic equipment, which includes an adult weighing scale, stadiometer, and digital & manual blood pressure apparatus, had a mean domain score of 56.4%. In the same vein, the health facility’s diagnostic capacity, which includes blood glucose test, urine protein test, and urine glucose test, had a mean domain score of 41.0%. The medicines for diabetes management had a mean domain score of 24.1%, which is relatively low compared to other domains. Overall, by taking all four domains into cognizance, the service readiness for providing diabetes care is 37.7%. Notably, the diabetes service readiness index specific to private for-profit facilities 22.3% is much lower compared to public 53.5%, private not-for-profit 43.0%, and mixed 48.4% health facilities, respectively.

 In addition, Table 4 presents the service readiness index for cardiovascular diseases and the scores of each domain. Of 7,606 health facilities that offer the services, only 1538 (23.7%) provide both diagnosis and management for cardiovascular diseases. Among the health facilities with service coverage for cardiovascular diseases, only 1174 (22.4%) had standard national guidelines for managing chronic cardiovascular diseases. Basic equipment and general medicines for managing cardiovascular diseases had a mean domain score of 65.4 and 18.6. Thus, this indicates that there is a low supply of drugs specific to managing cardiovascular diseases. Therefore, considering the three domains, the service readiness index for cardiovascular diseases is at 35.4.

 However, by health facility type, the readiness index is much lower for private for-profit health facilities at 19.7 compared to public health facilities at 51.6.

 Furthermore, Table 5 presents the service readiness index specific to respiratory diseases and its various domains. Of 5093 health facilities that offer the services, only 2139 (35.7%) diagnose and manage respiratory diseases. Similarly, only 1267 (24.5%) health facilities had national guidelines for managing chronic respiratory conditions. The mean domain score for equipment and medicines are 56.4 and 14.0. There is a notable shortage of supply of medicines for chronic respiratory diseases. It had the lowest mean domain score compared to the supply of medicines for diabetes at 24.1 and cardiovascular diseases at 18.6. While medicine for chronic respiratory diseases has a higher mean domain score for private for-profit health facilities, its scores are comparatively lower for diabetes and cardiovascular diseases domains. The service readiness index for chronic respiratory diseases across the three domains is 36.5. By health facility – type, akin to the scores of the domains for cardiovascular diseases, is obtained for chronic respiratory diseases.

## Discussion

 The focus of this study is to assess health facility services readiness for diabetes, cardiovascular, and respiratory diseases to better understand health facility services coverage for NCDs in LMICs. Assessing health facility services readiness for specific diseases with the highest mortality and morbidity burdens will provide insights into the prevailing deficits in health services coverage and inform policymakers on the urgent need for scale-up in investment in critical health infrastructure required to improve health outcomes in the UHC era. Thus, this study used cross-country data to estimate the general service and disease-specific readiness indexes in over 7000 health facilities. The mean scores of the general service readiness index revealed the fair readiness of health facilities to provide basic health services. Broadly, public health facilities achieved the highest scores in terms of service readiness, followed by profit-driven health facilities and private not-for-profit health facilities. While the service readiness index provides a list of items expected to be available in any health facility in line with the WHO recommendations, medical masks, eye goggles, and guidelines for standard precautions are notably in short supply. Similar to these results are documented in a cross-country analysis of service readiness by O’Neill et al.^16^

 Notably, the score of service readiness index for diabetes, cardiovascular, and respiratory diseases are quite low in the countries of this study. Generally, public facilities have achieved an above-average score of the service readiness index for the three chronic diseases. The profit-driven health facilities have outperformed private not-for-profit health facilities in the service readiness scores for cardiovascular and respiratory diseases. However, the exact opposite is obtained for diabetes service index scores between private for-profit and private not-for-profit health facilities. Notably, the supply of medicines for managing chronic diseases is quite low. It is particularly at the lowest supply for chronic respiratory diseases. This result is similar to the result reported by Biswas et al in the context of Bangladesh.^8^

 It is to be noted that private for-profit, mixed-owned, faith-based, and other types of health facilities have demonstrated lower services readiness index for the general and health services specific to diabetes, cardiovascular, and respiratory diseases. However, previous country-level studies conducted by Barber et al have shown that the public sector provides care of higher quality relative to the private sector.^17^ Identifying the difference in the quality of care received by individuals with chronic diseases between public and private health facilities is beyond the scope of the present paper. However, exploring the potential drivers of the general and services-specific readiness index may warrant further empirical investigations.

## Limitations

 However, this study is not without some limitations. For instance, in Afghanistan, the survey data covers only one region of the country unlike in other countries where the survey covers the entire regions or districts of the countries. The analysis found missing information on service provision for diabetes and respiratory diseases. The same is found in the items under general service readiness. Missing information on some items may lead to an underestimation of the service readiness index. Furthermore, the method section considers the studied countries as panel data without considering the heterogeneity in their composition. The study countries differ remarkably in composition, size, resource endowments, living standards, and their health system. Therefore, future studies should consider a country-level analysis and look into these disparities in the analysis of service coverage for these countries. Moreover, a lot has changed from the time the data is collected to date. As a result, the information reported may be different from what is currently obtainable in the study countries. Finally, a different perspective on the analysis of service readiness may produce evidence that may differ from what is currently reported due to some methodological errors. Therefore, a rigorous statistical test should be performed to choose the most suitable approach for the analysis of facility-level service readiness for general and disease-specific services to obtain robust estimates.

## Conclusion

 This study used cross-country facility-level data to examine the extent of general and disease-specific service readiness in 8 LMICS. The findings show a fair mean readiness index for the general services coverage. However, health facilities’ capacity to provide services for chronic diseases is relatively low. This demonstrated the existence of a grave deficit in health services coverage for chronic diseases in LMICs. This deficit must be urgently addressed if LMICs are to stay on track for achieving the goals of UHC. All efforts to improve health outcomes in the population will be impracticable if health facilities are not adequately equipped to provide care. Typically, the health systems of the countries of this study are characterized by a huge shortage of medicines for chronic diseases. Therefore, for these countries to improve health outcomes, efforts must be made with a specific focus on expanding health services coverage along with an adequate supply of medicines for people with underlying conditions.

## Policy Implications

 Nevertheless, this analysis of the general and disease-specific services readiness index has important policy suggestions. The existing deficit in health services coverage for chronic diseases could be narrowed by massive investment into the health systems to ensure wider availability of health services for people with underlying conditions from the primary, secondary, and tertiary levels of providing care in LMICs. Moreover, the level of investment in critical infrastructure required to achieve UHC goals cannot be sufficiently provided by the public sector alone. Thus, private sector and non – governmental organizations could also complement government efforts to improve health outcomes. Pursuing UHC goals through the demand side of care by mandating households to obtain health insurance would inevitably induce greater utilization of health services than without coverage. However, if health facilities are not adequately equipped with the supply of necessary equipment and skilled health providers to offer health services, the effect of obtaining coverage will not produce the desired outcomes.

## Competing Interests

 The authors declare that they have no known competing financial interests or personal relationships that could have appeared to influence the work reported in this paper.

## Ethical Approval

 Ethical approval for this type of study is not required by our University.

## Supplementary Files


Supplementary file 1 contains Table S1.

## References

[1] United Nations. Transforming our World: The 2030 Agenda for Sustainable Development. New York: United Nations; 2015. Available from: https://sustainabledevelopment.un.org/post2015/transformingourworld/publication. Accessed June 5, 2022.

[2] Savedoff WD, de Ferranti D, Smith AL, Fan V (2012). Political and economic aspects of the transition to universal health coverage. Lancet.

[3] Kruk ME, Gage AD, Joseph NT, Danaei G, García-Saisó S, Salomon JA (2018). Mortality due to low-quality health systems in the universal health coverage era: a systematic analysis of amenable deaths in 137 countries. Lancet.

[4] Donabedian A (1988). The quality of care. How can it be assessed? JAMA.

[5] Leslie HH, Spiegelman D, Zhou X, Kruk ME (2017). Service readiness of health facilities in Bangladesh, Haiti, Kenya, Malawi, Namibia, Nepal, Rwanda, Senegal, Uganda and the United Republic of Tanzania. Bull World Health Organ.

[6] Jigjidsuren A, Byambaa T, Altangerel E, Batbaatar S, Saw YM, Kariya T (2019). Free and universal access to primary healthcare in Mongolia: the service availability and readiness assessment. BMC Health Serv Res.

[7] Katende D, Mutungi G, Baisley K, Biraro S, Ikoona E, Peck R (2015). Readiness of Ugandan health services for the management of outpatients with chronic diseases. Trop Med Int Health.

[8] Biswas T, Haider MM, Das Gupta R, Uddin J (2018). Assessing the readiness of health facilities for diabetes and cardiovascular services in Bangladesh: a cross-sectional survey. BMJ Open.

[9] Daar AS, Singer PA, Persad DL, Pramming SK, Matthews DR, Beaglehole R (2007). Grand challenges in chronic non-communicable diseases. Nature.

[10] Yach D, Hawkes C, Gould CL, Hofman KJ (2004). The global burden of chronic diseases: overcoming impediments to prevention and control. JAMA.

[11] Lozano R, Naghavi M, Foreman K, Lim S, Shibuya K, Aboyans V (2012). Global and regional mortality from 235 causes of death for 20 age groups in 1990 and 2010: a systematic analysis for the Global Burden of Disease Study 2010. Lancet.

[12] World Health Organization (WHO). Noncommunicable Diseases Country Profiles 2014. Geneva: WHO; 2014. Available from: https://www.who.int/publications/i/item/9789241514620. Accessed July 13, 2018.

[13] El-Sayed AM, Palma A, Freedman LP, Kruk ME (2015). Does health insurance mitigate inequities in non-communicable disease treatment? Evidence from 48 low- and middle-income countries. Health Policy.

[14] The DHS Program. The DHS Program - Survey Search [Internet]. Available from: https://dhsprogram.com/methodology/survey-search.cfm?pgType = main&SrvyTp = type. Accessed October 9, 2021.

[15] World Health Organization (WHO). Service Availability and Readiness Assessment (SARA): An Annual Monitoring System for Service Delivery: Reference Manual. WHO; 2013. Available from: https://iris.who.int/handle/10665/149025.

[16] O’Neill K, Takane M, Sheffel A, Abou-Zahr C, Boerma T (2013). Monitoring service delivery for universal health coverage: the Service Availability and Readiness Assessment. Bull World Health Organ.

[17] Barber SL, Bertozzi SM, Gertler PJ (2007). Variations in prenatal care quality for the rural poor in Mexico. Health Aff (Millwood).

