# Supplementary material for: An analysis of health facility services readiness for non-communicable diseases in 8 LMICs in the universal health coverage era
Source: Health Promot Perspect. 2024 Dec 30;14(4):343–9. doi: 10.34172/hpp.43175 (PMC11873774; doi:10.34172/hpp.43175)
Supplement: Supplementary file 1 — contains Table S1. [file hpp-14-343-s001.pdf]

Singh et al, **Health Promotion Perspectives.** 2024;14(4):343-S1
doi: 10.34172/hpp.43175
https://hpp.tbzmed.ac.ir

Supplementary file 1

Table S1: Detail description of each service coverage domain

General Service Coverage	Service – Specific Coverage for Diabetes	Service – Specific Coverage for Cardiovascular Diseases	Service – Specific Coverage for Respiratory Diseases
Please indicate if this facility has the following functional infrastructure:<br>A. Power supply<br>B. Generator<br>C. Source of water<br>D. Communication equipment<br>E. Access to computer and internet<br>F. Emergency transportation services (ambulance)<br>Do you have the following equipment facility in this facility?<br>A. Digital blood pressure<br>B. Manual blood pressure<br>C. Adult scale<br>D. Child scale<br>E. Infant scale<br>F. Stethoscope<br>G. Thermometer<br>H. Light source<br>Standard Precautions and conditions for client examination<br>I. Sharps container (safety box).<br>J. Waste receptacle (pedal bin) with lid and plastic bin liner.<br>K. Running water (Piped, Bucket with tap or Pour Pitcher).<br>L. Hand washing soap (may be liquid soap).<br>M. Latex gloves.<br>N. Gowns.<br>O. Masks<br>P. Eye protection (goggles or face protection)<br>**Q.** Guidelines for standard precautions**.**<br>Do providers offer the following ***laboratory tests*** in this facility?<br>A. Haemoglobin<br>B. Blood glucose<br>C. Urine pregnancy tests<br>D. Syphilis	Do providers in this facility diagnose and/or manage ***diabetes***?<br>(a) Diagnose only<br>(b) Manage only<br>(c) Diagnose and manage<br>(d) No<br>Do you have the ***national guidelines*** for the diagnosis and management of diabetes available in this facility?<br>(a) Yes<br>(b) No<br>I would like to know if the following items are available today in the main service area and are functioning:<br>A. Adult weighing scale.<br>B. Stadiometer/Height board<br>C. Blood pressure<br>Are any of the following medicines for the management of ***diabetes*** available in this facility today?<br>A. Injectable insulin.<br>B. Glibenclamide<br>C. Metformin.<br>D. Injectable glucose solution.	Do providers in this facility diagnose and/or manage ***cardiovascular diseases***?<br>(e) Diagnose only<br>(f) Manage only<br>(g) Diagnose and manage<br>(h) No<br>Do you have the ***national guidelines*** for the diagnosis and management of ***cardiovascular diseases*** available in this facility?<br>(c) Yes<br>(d) No<br>I would like to know if the following items are available today in the main service area and are functioning:<br>A. Adult weighing scale.<br>B. Stadiometer/Height board<br>C. Blood pressure<br>Are any of the following medicines for the management of ***cardiovascular diseases*** available in this facility today?<br>A. Amlodipine/nifedipine.<br>B. Atenolol (Beta-blockers).<br>C. Captopril.<br>D. Nifedipine tablet.<br>E. Thiazide.	Do providers in this facility diagnose and/or manage ***respiratory diseases***?<br>(i) Diagnose only<br>(j) Manage only<br>(k) Diagnose and manage<br>(l) No<br>Do you have the ***national guidelines*** for the diagnosis and management of ***respiratory diseases*** available in this facility?<br>(e) Yes<br>(f) No<br>I would like to know if the following items are available today in the main service area and are functioning:<br>A. Adult weighing scale.<br>B. Stadiometer/Height board<br>C. Blood pressure<br>Are any of the following medicines for the management of ***respiratory diseases*** available in this facility today?<br>A. Salbutamol inhaler<br>B. Simvastatin<br>C. Beclomethasone

Source: *World Health Organization*
